# Effect of scenario-supported training on pediatric nurses' knowledge and skills in extravasation and infiltration management

**DOI:** 10.3389/fped.2025.1734531

**Published:** 2026-01-14

**Authors:** Emel Uyar, Bilge Delibalta, Nese Ozyurt

**Affiliations:** 1 Department of Pediatrics, University of Health Sciences, Ankara Bilkent City Hospital, Ankara, Türkiye; 2 Department of Medical Education and Informatics, Faculty of Medicine, Hacettepe University, Ankara, Türkiye; 3 Department of Pediatric Nursing, Faculty of Nursing, Yıldırım Beyazıt University, Ankara, Türkiye

**Keywords:** extravasation, infiltration, nursing education, OSPE, pediatric nursing, scenario-based training, simulation

## Abstract

**Background:**

Extravasation and infiltration are preventable complications of intravenous therapy that can cause severe tissue injury in pediatric patients. Nurses play a critical role in prevention and early management, yet studies have shown that their knowledge and skills in this area are often insufficient. Simulation-based training has been shown to improve clinical competence; however, evidence specific to pediatric extravasation management is limited.

**Methods:**

This quasi-experimental study employed a single-group pretest–posttest design. A total of 475 pediatric nurses were invited, 436 participated, and 399 were included in the final analysis after missing data excluded. A half-day, scenario-supported training program was developed by expert clinicians, combining theoretical lectures with low-fidelity, case-based simulation activities. Pre- and post-training scores were compared using nonparametric tests, with significance set at *p* < 0.05 and effect sizes reported. Knowledge was assessed using a validated 55-item test, and skills were measured through a four-station ObjectiveStructured Practical Examination (OSPE).

**Results:**

Participants were predominantly female (84.7%) and held a bachelor's degree (87.5%). The majority (77.9%) had never received prior extravasation training, while 88.5% expressed a need for education. Knowledge scores improved significantly post-training (*p* < 0.001, *r* = –0.26, small effect). Skill scores showed a marked improvement (*p* < 0.001, *r* = –0.84, large effect). Subgroup analyses revealed no significant differences by gender or educational level. Nurses with 5–8 years of experience demonstrated greater knowledge gains, while those with prior training achieved higher skill improvements.

**Conclusion:**

Scenario-supported training effectively improved pediatric nurses' competencies in extravasation and infiltration management, with the strongest effect observed in clinical skills. These findings underscore the importance of structured and repeated training, particularly for early- and mid-career nurses. Low-cost, scalable training models represent a practical and sustainable strategy to strengthen pediatric nursing practice and enhance patient safety in diverse healthcare settings.

## 1 Introduction

Intravenous therapy is essential in pediatric care but carries the risk of serious complications. Among these, infiltration and extravasation can lead to tissue necrosis, functional impairment, and cosmetic sequelae (1, 2). Infiltration refers to the leakage of non-vesicant solutions, whereas extravasation involves vesicant or irritant agents leaking into surrounding tissues (3). Due to their immature skin, fragile vasculature, and limited ability to communicate symptoms, pediatric patients are at higher risk compared with adults (4, 5). Reported incidence rates vary widely, ranging from 10% to 46%, underscoring extravasation as a preventable patient safety concern (6–8).

The preventable nature of these complications highlights the critical role of pediatric nurses in early recognition and effective management. However, several studies indicate that nurses' knowledge and practices regarding extravasation remain insufficient. Moreover, many nurses report never having received structured training and consistently express the need for further education (9–11).

Simulation-based training provides a safe and repeatable environment where nurses can practice clinical reasoning and procedural skills without endangering patients. Systematic reviews have shown that simulation enhances clinical performance and decision-making (12–15). Yet, only a few interventional studies have specifically addressed extravasation management. For example, Taylor (16) reported improvements in pediatric intravenous site care following an evidencebased educational project. High-fidelity simulation studies have further shown that nursing students' competency in managing chemotherapy extravasation can be significantly improved (17). In neonatal care, the implementation of clinical practice guidelines has been associated with better prevention and management of extravasation injuries (18).

Despite these encouraging findings, the available evidence is either highly specialized (oncology, neonatology) or resource-intensive (high-fidelity simulation). Evidence on structured, scenario-supported training for pediatric nurses in general clinical settings remains scarce. Thus, there is a need for low-cost, scalable educational approaches that can be integrated into routine pediatric nursing practice.

This study hypothesizes that scenario-based extravasation and infiltration management training will result in significant improvements in pediatric nurses' knowledge and procedural skills pertaining to the recognition, intervention, and management of extravasation. Accordingly, the aim of the study was to evaluate the effect of a scenario-supported training program on pediatric nurses' knowledge and skills in extravasation and infiltration management.

## 2 Materials and methods

### 2.1 Study design and participants

This study was designed as a single-group pretest–posttest quasi-experimental intervention. It was conducted between June and September 2025 at the pediatric clinics of Ankara Bilkent City Hospital, Türkiye. Eligible participants were nurses actively working in pediatric wards for at least six months who provided written informed consent. Nurses who declined participation or did not complete the data collection procedures were excluded.

A total of 475 nurses were employed at Ankara Bilkent City Hospital, all of whom were invited to participate in the study. A formal sample size calculation was not performed; instead, the study sought to recruit at least 80% of the nursing workforce. Of these, 436 nurses agreed to participate (91.8%). Following the exclusion of incomplete responses and extreme values, data from 399 nurses were retained for the final analysis. Ethical approval was obtained from the Ankara City Hospital Clinical Research Ethics Committee (Approval No: TABED-1-25-1297).

### 2.2 Training program and data collection tools

#### 2.2.1 Demographic information form

The Demographic Information Form collected data on participants' age, gender, education level, professional experience, previous exposure to extravasation cases, and prior training related to intravenous therapy and extravasation management.

#### 2.2.2 Development of the training and assessment framework

Both the training program and the assessment tools were developed by a multidisciplinary study team consisting of one pediatric intensive care specialist, one wound care nurse, two nurse educators, one senior nurse, and one medical educationist, and were structured in accordance with the Institutional Protocol for Extravasation and Infiltration Management (Supplementary material 1) and current literature (3, 4, 6, 9, 10, 16, 18, 19). During the development of the training and assessment framework, a blueprint was created to align the learning outcomes, teaching methods, and assessment strategies with contemporary educational standards and current literature (20). Two independent experts reviewed the blueprint and all assessment items to ensure content validity, relevance, and congruence with the intended learning outcomes. The blueprint of the training is shown in Supplementary Table S1. The program consisted of a half-day (4 h) session delivered in groups of 25 nurses (19 sessions in total). Training included a combination of theoretical lectures and scenariobased practice. Simulation was applied at a low-fidelity, scenario-supported level, primarily in the form of case-based discussions and structured clinical examinations. No high-fidelity manikins or advanced simulation technologies were used.

#### 2.2.3 Knowledge test

The knowledge test was constructed based on current scientific evidence and clinical practice guidelines related to extravasation and infiltration management (3, 4, 6, 9, 10, 16, 18, 19). The instrument consisted of 55 true/false and multiplechoice items, each worth one point, yielding a total score of 55.

Content validity was assessed by three independent experts in pediatric nursing and medical education, who examined each item for clarity, relevance, and alignment with the learning objectives. Necessary revisions were made based on expert feedback. Internal consistency reliability, evaluated using Cronbach's alpha, demonstrated acceptable reliability (*α* = .768), indicating adequate interrelatedness among items and coherence within the construct measured.

#### 2.2.4 Skill test: objective structured practical examination (OSPE)

Practical skills were evaluated using an Objective Structured Practical Examination (OSPE), a structured and standardized method originally developed by Harden and Cairncross (21) to assess clinical and procedural competencies. The OSPE consisted of four stations:

• Peripheral IV Infiltration
• Early recognition of extravasation (Vesicant Drug Extravasation Identification)
• TPN Extravasation Intervention
• Compartment Syndrome Assessment & Monitoring.

Each station included a checklist with observable performance criteria. The initial 45-item OSPE checklist was developed by the study team based on key extravasation management literature (3, 4, 6, 9, 10, 16, 18, 19) and subsequently reviewed by two external experts for content and face validity. Following expert review, two redundant items were removed, resulting in a final 43-item checklist. Each item was scored as one point, yielding a maximum OSPE score of 43.

The OSPE was designed in accordance with established principles of objectivity, structure, transparency, and standardization (21, 22). The explicit checklist format minimized examiner bias and improved reliability. The internal consistency reliability of the knowledge assessment instrument and the OSPE checklist was rigorously evaluated utilizing Cronbach's alpha coefficient. Internal consistency was moderate (*α* = .532), an acceptable value for performance-based assessments that evaluate multiple discrete skills rather than a single latent trait.

#### 2.2.5 Scoring and administration procedures

The combined maximum score for both instruments was 98. Pretests were administered immediately before the training, and posttests were conducted immediately after completion of the training program.

### 2.3 Statistical analysis

Data were analysed using IBM SPSS Statistics version 26 (IBM Corp., Armonk, NY, USA). Missing values and outliers were screened using the interquartile range method. The Kolmogorov–Smirnov test indicated non-normal distribution of continuous variables; therefore, nonparametric tests were applied.

• Wilcoxon signed-rank test was used to compare pretest and posttest scores.
• Mann–Whitney *U* test was used for two independent group comparisons.
• Kruskal–Wallis test was applied for comparisons across three or more groups, with posthoc analyses performed where significant differences were found.

Descriptive statistics are presented as median (minimum-maximum) for continuous variables and frequency (*n*, %) for categorical variables. A *p*-value < 0.05 was considered statistically significant. Effect sizes (*r*) were also calculated and reported.

## 3 Results

### 3.1 Participant characteristics

Of the 475 nurses invited to the program, 436 agreed to participate (91.8%). After excluding incomplete and outlier data, analyses were performed on 399 participants. The majority were female (84.7%, *n* = 338), and the median age was 28 years (range: 23–53). Most participants held a bachelor's degree (87.5%, *n* = 349) and nearly half had 5–8 years of professional experience (47.4%, *n* = 189).

Regarding prior exposure to extravasation, 8.3% (*n* = 33) reported never encountering a case, 77.7% (*n* = 310) rarely, and 14% (*n* = 56) frequently. Most participants (77.9%, *n* = 311) had never received any training on extravasation, while 88.5% (*n* = 353) expressed a need for further education.

### 3.2 Knowledge and skill scores

Knowledge test scores significantly increased following training (*p* < 0.001), with a small effect size (*r* = –0.26). Skill test scores also improved significantly (*p* < 0.001), with a large effect size (*r* = –0.84), indicating substantial gains in clinical performance. General characteristics of the test results and the comparison of the mean scores are summarized in Tables 1, 2.

**Table 1:** Distribution of pretest and posttest scores for knowledge and skill assessments.

Test	Mean	SD	Median	Minimum	Maximum
Knowledge Pretest	39.31	5.77	40	22	51
Knowledge Posttest	40.84	4.35	42	16	53
Skill Pretest	32.44	3.32	32	34	42
Skill Posttest	38.56	2.71	39	30	51

**Table 2:** Comparison of knowledge and skill pretest and posttest scores (Wilcoxon SignedRank test).

Compared parameters	*n*	Mean Rank	Sum of Ranks	Z	*p*
Knowledge Test	399			−5.138	**0** **.** **000** *
Negative Ranks	148	165.39	24,477.00		
Positive Ranks	227	202.74	46,023.00		
Ties	24				
Skill Test	399			−16.748	**0** **.** **000** *
Negative Ranks	12	41.46	497.50		
Positive Ranks	371	196.87	73,038.50		
Ties	16				

Bold values indicate statistically significant results (*p* < 0.05). Values displayed as 0.000 should be interpreted as *p* < 0.001.

* The result was statistically significant at *p* < 0.05.

The results of the subgroup analyses are presented in Table 3.

• Gender: No significant differences were observed in pretest scores between male and female nurses. Posttest improvements were higher among female participants, but the difference was not statistically significant (*p* > 0.05).
• Educational Level: No significant differences were found between nurses with different educational backgrounds (associate, bachelor's, master's, doctorate) in terms of pretest– posttest score changes (*p* > 0.05).
• Professional Experience: Skill score improvements did not differ by years of experience (*p* > 0.05). However, knowledge score improvements were significantly higher among nurses with 5–8 years of experience compared to other groups (*p* < 0.05).
• Prior Training: Nurses who had previously received extravasation training had higher baseline knowledge and skill scores (*p* < 0.05). In posttest comparisons, no difference was found in knowledge score improvements (*p* > 0.05), but skill score improvements were significantly greater among those with prior training (*p* < 0.05, *r* = 0.03, small effect size).

**Table 3:** Subgroup analysis of knowledge and skill scores by demographic and training variables.

Compared Variable	Difference Between Knowledge Posttest and Pretest Scores		Difference Between Skill Posttest and Pretest Scores	
	*n*	*P*	*n*	*p*
Gender		0.139		0.464
Female	338		338	
Male	61		61	
Age		0.812		0.824
<25 years	69			
26–29 years	234			
30–39 years	81			
>40 years	15			
Education Level		0.574		0.693
Associate Degree	4			
Bachelor's Degree	349			
Master's Degree	31			
Doctorate (PhD)	1			
Years of Professional Experience		0.020*		0.872
<1 year	56			
2–4 years	102			
5–8 years	189			
>9 years	52			
Prior Training Status				
No	311	0.037*	311	0.218
Yes	88		88	

* Significant difference was found between the two groups (*p* < 0.05).

## 4 Discussion

### 4.1 Effects on knowledge and skills

This study evaluated the impact of scenario-supported extravasation and infiltration management training on pediatric nurses' knowledge and skills. Findings showed a small effect on knowledge (*r* = –0.26) and a large effect on skills (*r* = –0.84), indicating that the program was particularly effective in improving practical competencies. Previous research supports these results, showing that simulation-based education significantly enhances clinical reasoning and performance (12, 14). Our results highlight that experiential, scenario-based approaches may facilitate the transfer of knowledge to clinical practice more efficiently than traditional didactic methods, especially in the development of procedural and decision-making skills in line with the literature. Also, our study reinforces existing evidence by showing that structured, scenario-driven training yields greater improvements in skill-based outcomes compared to theoretical knowledge gains.

### 4.2 Gender and professional experience

Although female nurses achieved greater improvements in both knowledge and skill scores than male nurses, these differences were not statistically significant. This is consistent with studies reporting that simulation-based learning benefits learners regardless of gender (23). Accordingly, the scenario-based low-fidelity simulation training appears to be equally effective for nurses of both genders.

Professional experience was found to influence knowledge improvement, particularly among nurses with 5–8 years of work experience. According to Benner's “From Novice to Expert” model, these nurses are typically in the *competent* or *proficient* stages of practice, where structured training can accelerate professional development (24, 25). In contrast, novice nurses may require additional foundational support, while more experienced nurses may approach a performance ceiling. These findings suggest that mid-career nurses are especially positioned to benefit from scenario-based education and that considering professional stage when forming training groups may optimise educational outcomes.

### 4.3 Need for repeated training

A notable finding was that 78% of participants had never received previous extravasation training, and 88% expressed a strong need for education, indicating a substantial gap in routine clinical training practices. Moreover, nurses with prior training demonstrated greater skill improvements, even though knowledge improvements did not differ. This suggests that repeated and practice-based training plays a critical role in reinforcing competencies. Consistent with earlier studies, repeated exposure to simulation has been shown to promote mastery and self-efficacy (13, 15). Given the potential severity of extravasation events in pediatric care, this lack of structured education represents a preventable patient-safety risk. Therefore, these findings emphasize the necessity of embedding recurring scenario-based modules into continuous professional development instead of relying on one-time programs.

### 4.4 Comparison with previous studies and clinical implications

Similar positive outcomes have been reported in oncology nursing. For example, educational interventions on safe chemotherapy administration improved knowledge and skills among nurses (26). High-fidelity simulation has also been shown to significantly enhance nursing students' competence in managing chemotherapy extravasation (27). In neonatal care, guideline implementation studies demonstrated improvements in the prevention and management of extravasation injuries (18). Our study contributes to this evidence by demonstrating that a low-cost, scenario-supported training model can achieve meaningful skill gains among practicing pediatric nurses. While highfidelity simulation remains valuable, its resource requirements may limit widespread adoption; therefore, our findings indicate that simplified scenario-based approaches offer a scalable and accessible alternative, particularly for institutions with constrained resources. Moreover, the clinical applicability of such models suggests that structured and recurring scenario-based programs should be incorporated into pediatric nursing curricula, with emphasis on practical skill development and cost-effective implementation strategies.

### 4.5 Strengths and limitations

Strengths of this study include the large sample size (*n* = 399), the structured design using both pre- and post-tests, and the combined evaluation of knowledge and skills. However, several limitations should be acknowledged. First, the study was conducted at a single center with a predominantly young and bachelor-level nurse population, limiting generalizability. Second, the effects were assessed only in the short term; long-term knowledge and skill retention remain unknown. Third, no randomized controlled design was used, which reduces the strength of causal inference. As expected in performance-based assessments, the OSPE demonstrated moderate internal consistency. OSPE checklists typically include diverse procedural steps that do not necessarily correlate strongly with one another, which can lower alpha coefficients without compromising the validity of the assessment (21). Despite this, the structured checklist and standardized scoring procedure enhance objectivity and reduce examiner bias, supporting the practical reliability of the tool. Finally, simulation in this program was low-fidelity and primarily applied as an assessment method through case-based scenarios and OSPE, rather than as immersive high-fidelity training. This may explain the relatively modest improvement in knowledge scores.

## 5 Conclusion

This study demonstrated that scenario-supported extravasation and infiltration management training improved pediatric nurses' competencies, with a particularly strong effect on practical skills compared to theoretical knowledge. The findings highlight the importance of structured and repeated training to reinforce knowledge retention and enhance skill acquisition. Considering that most nurses had never received prior training but expressed a strong need for it, such programs should be prioritized, especially for early- and mid-career nurses. Importantly, the use of low-cost and scalable scenario-based approaches offers a feasible and sustainable strategy to improve patient safety and quality of care in pediatric settings, particularly in resource-limited environments.

## Data Availability

The original contributions presented in the study are included in the article/Supplementary Material, further inquiries can be directed to the corresponding author.

## Supplementary material

Supplementary Table S1 Learning outcomes, teaching methods, and assessment alignment based on Bloom’s taxonomy.
