# Supplementary material for: Effect of scenario-supported training on pediatric nurses' knowledge and skills in extravasation and infiltration management
Source: Front Pediatr. 2026 Jan 14;13:1734531. doi: 10.3389/fped.2025.1734531 (PMC12847387; doi:10.3389/fped.2025.1734531)
Supplement: Supplementary Table S1 — Learning outcomes, teaching methods, and assessment alignment based on Bloom’s taxonomy. [file Table1.docx]

**Supplementary Table 1.** Learning outcomes, teaching methods, and assessment alignment based on Bloom’s taxonomy.

| **Learning outcome** | **Teaching method** | **Assessment method** |
| --- | --- | --- |
| The learner explains the concepts of infiltration and extravasation and lists key differences between them. | Theoretical lecture | Knowledge Test |
| The learner identifies vesicant and non-vesicant medications and classifies them with examples. | Theoretical lecture, drug flashcards | Knowledge Test |
| The learner describes risk factors associated with extravasation. | Theoretical lecture | Knowledge Test |
| The learner explains peripheral IV complications and correlates them with clinical signs and symptoms. | Theoretical lecture, case discussion | Knowledge Test |
| The learner recalls and sequences the steps of the institutional extravasation protocol. (Supplementary material 1) | Theoretical instruction, institutional protocol review | Knowledge Test |
| The learner applies observation criteria for early recognition of infiltration/extravasation during IV therapy. | Case-based scenarios, interactive discussion | OSPE |
| The learner evaluates and applies appropriate interventions for vesicant extravasation, including antidotes and temperature therapies. | Case-based scenarios, interactive discussion | OSPE |
| The learner documents extravasation correctly, including written records and photographic documentation. | Case-based scenarios, interactive discussion | OSPE |
| The learner formulates a monitoring and follow-up plan | Case-based scenarios, interactive discussion | OSPE |
| The learner assesses early signs of compartment syndrome and determines appropriate nursing interventions. | Case-based scenarios, interactive discussion | OSPE |
