# Supplementary material for: Effect of scenario-supported training on pediatric nurses' knowledge and skills in extravasation and infiltration management
Source: Front Pediatr. 2026 Jan 14;13:1734531. doi: 10.3389/fped.2025.1734531 (PMC12847387; doi:10.3389/fped.2025.1734531)
Supplement: Supplementary file 2 [file Datasheet1.docx]

**Supplementary material 1**. Institutional Protocol for Extravasation and Infiltration Management

**1. Recognition & Initial Response**

- 1. Extravasation should be suspected when any of the following occur:

• pain, burning, or discomfort at the infusion site

• swelling, blanching, erythema, or coolness

• resistance during infusion

• leakage around insertion site

• vesicant drug infusion with unusual patient reaction

- 1. Differentiate:

• Infiltration: non–vesicant solution leakage

• Extravasation: vesicant or irritant drug leakage

**2. Immediate Actions**

2.1 Stop the infusion

• Stop infusion immediately.

• Do not flush the cannula.

2.2 Aspirate

• Attempt to aspirate residual drug using a 10 mL syringe.

• Do NOT apply pressure or massage.

2.3 Cannula management

• Keep the cannula in place during aspiration.

• Do not remove the cannula until the need for an antidote has been assessed.

• The cannula should be removed after photographic documentation is obtained and the need for an antidote is clarified.

• If an antidote is required, administration through the existing cannula is preferred when possible.

2.4 Avoid pressure & manipulation

• Do not massage.

• Avoid compression or squeezing.

• Do not apply heat/cold unless drug-specific protocol requires.

2.5 Mark the affected area

• Outline the border.

• Date and timestamp marking.

**3. Notify & Initiate Treatment**

3.1 Notify

• Notify the responsible physician immediately.

• Consult surgical services and the clinical pharmacist, if needed.

3.2 Administer antidote

• As per drug classification:

• vesicant

• irritant

• non-vesicant

• Follow institutional guidelines.

3.3 Limb elevation

• Elevate extremity.

• Promote venous return.

3.4 Analgesia

• Administer pain relief as necessary.

• Evaluate effect.

**4. Documentation Requirements**

**The following MUST be recorded:**

4.1 Drug Information

• Drug name

• Concentration

• Total volume infused

• Estimated volume extravasated

• Vesicant vs irritant vs non-vesicant classification

• Antidote administered (name, dose, time)

4.2 Infusion Details

• Cannula type and gauge

• Site of insertion

• Dwell time

• Infusion method (bolus or continuous)

• Pump settings (if applicable)

4.3 Clinical Site Assessment

• size and diameter of swelling

• skin temperature, color, firmness

• capillary refill time

• distal pulses

• pain severity

• sensory changes (paresthesia)

4.4 Photographic Documentation

• Take an immediate photograph of the affected site.

• Capture serial images to monitor progression.

• Ensure all images include date and time stamps.

• Upload photographs to the hospital’s electronic medical record (EMR) system.

4.5 Communication

• physician notified

• specialist consulted

• education delivered to patient/family

**5. Compartment Syndrome Monitoring**

Serial monitoring must evaluate:

5.1. Early signs:

• disproportionate pain

• tense, firm swelling

• paresthesia

5.2. Progression:

• pallor

• decreased capillary refill

5.3. Late signs (urgent):

• pulselessness

• paralysis

5.4. Documentation should include:

• time–interval assessments

• escalation steps

**Immediate surgical consultation if suspected.**

**6. Patient & Family Communication**

Nurse must:

• inform family of event

• explain risks and treatment plan

• instruct on follow-up signs (worsening pain, color change, swelling)

**7. Follow-Up & Outcome Tracking**

7.1. Continue monitoring until:

• resolution

• referral

• surgical intervention (if needed)

7.2. Record:

• clinical progression

• photographic comparison

• final outcome

**8. Institutional Quality & Reporting**

• Record incident in institutional reporting system.

• Review event for prevention and education.

• Use for staff training and protocol reinforcement.
